# “It Was the Best Decision of My Life”: a thematic content analysis of former medical tourists’ patient testimonials

**DOI:** 10.1186/1472-6939-16-8

**Published:** 2015-01-22

**Authors:** Carly Hohm, Jeremy Snyder

**Affiliations:** Faculty of Health Sciences, Simon Fraser University, Blusson Hall 10516, 8888 University Drive, Burnaby, BC V5A 1S6 Canada

**Keywords:** Medical tourism, Informed decision-making, Patient testimonials

## Abstract

**Background:**

Medical tourism is international travel with the intention of receiving medical care. Medical tourists travel for many reasons, including cost savings, limited domestic access to specific treatments, and interest in accessing unproven interventions. Medical tourism poses new health and safety risks to patients, including dangers associated with travel following surgery, difficulty assessing the quality of care abroad, and complications in continuity of care. Online resources are important to the decision-making of potential medical tourists and the websites of medical tourism facilitation companies (companies that may or may not be affiliated with a clinic abroad and help patients plan their travel) are an important source of online information for these individuals. These websites fail to address the risks associated with medical tourism, which can undermine the informed decision-making of potential medical tourists. Less is known about patient testimonials on these websites, which can be a particularly powerful influence on decision-making.

**Methods:**

A thematic content analysis was conducted of patient testimonials hosted on the YouTube channels of four medical tourism facilitation companies. Five videos per company were viewed. The content of these videos was analyzed and themes identified and counted for each video.

**Results:**

Ten main themes were identified. These themes were then grouped into three main categories: facilitator characteristics (e.g., mentions of the facilitator by name, reference to the price of the treatment or to cost savings); service characteristics (e.g., the quality and availability of the surgeon, the quality and friendliness of the support staff); and referrals (e.g., referrals to other potential medical tourists). These testimonials were found either not to mention risks associated with medical tourism or to claim that these risks can be effectively managed through the use of the facilitation company. The failure fully to address the risks of medical tourism can undermine the informed decision-making of potential medical tourists, particularly given the considerable influence on decision-making by patient testimonials.

**Conclusions:**

Regulation of these global companies is difficult, making the development of testimonials highlighting the risks of medical tourism essential. Additional research is needed on the impact of patient testimonial videos on the decision-making of potential medical tourists.

## Background

Medical Tourism is understood as international travel with the intent of obtaining medical care, that is paid for out-of-pocket, and beyond the scope of government-administered, cross-border care arrangements. Individuals opt to engage in medical tourism for many reasons that are influenced by the individual’s health needs, the country from which they are travelling, their financial status, and their comfort and familiarity with international travel. Individuals are motivated to travel abroad for care due to price differences for care delivered abroad compared to at home, the unavailability of treatment in the home country due to lengthy wait-times for limited resources, and the desire to access unproven treatments which are not available in the patient’s home country [1].

While medical tourism provides safe and timely access to care for many of its users, going abroad for care may also entail new risks for participants. Scholars researching this topic have raised concerns around several potential new risks. Flying with a serious medical condition or flying post-operatively may increase a patient’s risk of developing deep vein thrombosis [2]. Patients receiving medical care abroad may also be at an increased risk of contracting an infection in the facilities abroad and having post-operative strains on their recovery as a result of tourist activities or travelling back to their home country [3]. In addition, medical tourism can undermine continuity of care as care is administered by medical professionals internationally and medical records are often not transferred between the patient’s home physician and the health care professional abroad [4]. For some patients, obtaining aftercare upon returning to their home country can be difficult. In rare cases, patients may face barriers in finding doctors to administer care to them upon their return as they do not want to be held legally responsible for complications that may arise [5]. Moreover, should complications arise, the patient may have problems communicating with the facility abroad where the care was administered [6].

There are many methods by which the individual may learn about medical tourism and choose whether and where to engage in this practice. Research with previous medical tourists from Canada has identified four main methods that individuals use to learn about medical tourism. These include “word-of-mouth, non-targeted internet searches, print and televised media stories and advertising, and familiarity with other countries’ health systems due to their having emigrated from them” (pg. 5) [7]. Those individuals seeking information via internet searches will encounter medical tourism facilitation companies prominently in their search results. Medical tourism facilitators are akin to travel agents who help to guide people seeking treatment abroad to specific facilities. They engage in a range of activities, including arranging travel and booking procedures to guiding medical decision-making [8]. Their income sources are often not transparent and include referral payments from international clinics, raising concerns about conflicts of interest [9, 10]. Medical tourism facilitator websites are an important source of online information about medical tourism and are often a first point of contact for people researching this practice [7, 11, 12]. These facilitator websites employ a vast array of advertising techniques in order to entice prospective medical tourists to make use of these services. Examinations of the information contained in medical tourism facilitator websites have indicated that these websites typically oversell the benefits of medical tourism and fail to report the risks associated with this practice [13, 14]. While not surprising, this information bias is problematic as it undermines the ability of individuals considering engaging in medical tourism to make informed choices about their medical care if this information is not augmented by less biased sources [15].

One of the persuasive tactics often used on these websites is the inclusion of videos of patient testimonials, in which patients relay first-hand accounts of the medical care they received while abroad [13]. While facilitator websites hosting these videos have been studied [11, 13, 14, 16], the content of these testimonial videos has not been analyzed. A better understanding of the content of these videos is important to understanding the impact of industry information sources on the decision-making of medical tourists as first person testimonial videos have been found to be extremely influential on health-related decision-making [17–19]. Individuals tend to give a great deal of credence to first-hand accounts when making important health-related decisions for themselves [20, 21]. The perceived credibility of the speaker appears to have significant impact on the credibility of the message being portrayed in the testimonial, especially when medical decisions are involved. A prospective patient is much more likely to be swayed by a testimonial being delivered if the patient can find some degree of relatedness to the speaker, including sharing a medical condition, as will be likely in the context of individuals seeking specialized medical care abroad [20]. Moreover, interviews with former medical tourists have found that patients appreciate and greatly value narrative first-hand accounts from other former medical tourists. Having access to other medical tourists helps to provide a support network from which prospective patients can obtain advice and a sense of belonging with others who have experienced similar circumstances [7].

This paper examines the content of patient testimonials on facilitator websites using a thematic content analysis to assess the major themes present in testimonial videos on facilitator webpages. The major themes, identified through grouping as part of the content analysis, are discussed with regard to their ethical significance and their potential impacts on the medical decision-making process. We argue that patient testimonial videos created and hosted by medical tourism facilitation companies convey a one-sided message that may impact the prospective patient’s ability to make a truly informed choice regarding engaging in medical tourism.

## Methods

In order to determine the main emerging themes expressed in patient testimonial videos hosted by medical tourism facilitation companies, a thematic content analysis was undertaken of videos posted on the popular internet search engine YouTube. A thematic content analysis is generally undertaken when one wishes to determine the frequency of a given theme in some aspect of research [22]. These videos were also hosted on the websites of facilitation companies and these websites linked to the respective YouTube channels. Focus on these videos, as opposed to others filmed by former medical tourists in which there is no apparent facilitator presence, allows insight into the industry perspective in these patient testimonial videos.

The search term “medical tourist testimonial” was used to obtain links to a sample of patient testimonial videos available on YouTube. From this initial list, only testimonials published on medical tourism facilitator channels were considered. Four main facilitator channels were identified as popular publishing venues of testimonial videos based on their view rankings on YouTube. The four facilitator pages included Medical Tourism in Europe, Angels Abroad, Lotus Medical International (LMI), and Medical Tourism Corporation.

These four facilitator companies present a diverse sampling of patient testimonial videos for procedures delivered throughout the world. Medical Tourism in Europe is part of the facilitating company Wellness-Travels and focuses primarily on sending patients to medical facilities in Lithuania [23]. Angels Abroad primarily sends patients to Guatemala and offers a range of procedures including cosmetic surgery, essential care such as cardiovascular care and gastrointestinal care, and dental care [24]. Lotus Medical International operates out of Thailand and primarily offers plastic surgery procedures to foreign patients [25]. Finally, Medical Tourism Corporation operates out of the United States and organizes medical, cosmetic and dental procedures for patients throughout the world [26].

To conduct the analysis, five videos were selected and analyzed per facilitator page with the expectation that 20 total videos would allow for saturation of major themes, as was later confirmed. Content from the videos was summarized and main themes were identified through grouping as part of the content analysis and noted from each video. A frequency estimate of each main theme extracted from the videos was calculated based on the number of videos that explicitly mentioned the main theme and was verified by both authors. These findings and a frequency calculation of total mentions of themes within the videos are included in Table 1.

**Table 1:** **Summary of emerging themes**

Theme number	Main emerging theme	Number of videos referring to this theme	Total frequency of mentions of themes
**1**	Quality and availability of surgeon or other health care professional (e.g. Dentist)	17	42
**2**	Quality and Friendliness of Support Staff (e.g. Nurses, Front Desk, etc.)	14	22
**3**	Mentioned Surgeon or other main Health Care Professional by name	7	12
**4**	Reference to Facilitator Company they may have used:	18	44
**4a**	a.) Appreciation toward company	12	16
**4b**	b.) Reference to representative/guide’s help	8	25
**4c**	c.) Company took care of everything	10	25
**5**	Sense of Security/ Worry taken away/All Questions answered	11	35
**6**	Reference to Quality and Expediency of Care	14	34
**7**	Price of Procedure/Cost savings	13	21
**8**	Impacts of Surgery on Holiday/Tourist Activities	4	7
**9**	Reference to general cleanliness and high-tech services provided by the hotel/accommodations and the clinic/hospital	16	32
**10**	Would recommend to prospective Medical Tourists	14	19

## Results

From the twenty videos examined in the analysis, ten main themes were identified. Main themes included: 1) the quality and availability of the surgeon and other main health care professionals; 2) the quality and friendliness of the support staff; 3) specific reference to the surgeon that performed the procedure; 4) specific reference to a facilitator company that may have been used^a^; 5) a general sense of security and that worries were alleviated; 6) explicit reference to the quality and expediency of care; 7) reference to the price and cost savings of the care; 8) the impacts of the surgery on holiday and tourist activities; 9) reference to the cleanliness and services provided; and 10) whether they would recommend medial tourism as an option to future prospective patients. Table 1 provides a summary of the emerging themes, the number of videos referring to the theme, and the total frequency of mentions of the themes within the videos.

All of the patient testimonial videos assessed in this thematic content analysis presented a strictly positive view of medical tourism as a health care alternative. In addition, any negative aspects of the experience, such as feelings of nervousness on the day of the procedure, were explained away as normal feelings when undergoing major surgery or it was noted that they were mitigated by the facility staff or the facilitator agency representative.

Once frequency values were calculated, the majority of the main emerging themes were observed as appearing at very high frequency within the videos. Those that did not appear at high frequency included 1) mentioning the surgeon or health care professional by name; 2) that all their questions were answered and worries were taken away; and 3) any impacts the surgery had on their holiday and tourist activities. The themes that did appear at high frequency are grouped into three main categories: facilitator characteristics; service characteristics; and referrals.

The first category focuses on facilitator characteristics, including mentions of the facilitator by name in the testimonial and reference to the price of the treatment or to cost savings. Reference to a facilitation company was apparent in 18 out of the 20 videos examined with a total frequency of 44 mentions. Reference to cost savings was apparent in 13 out of the 20 videos analyzed with a total frequency of 21 mentions. When these points were mentioned in the testimonials, the speakers were enthusiastic in their portrayals of these messages and these points were often repeated more than once. One speaker proclaimed “if it wasn’t for Angels Abroad [the facilitator company] I wouldn’t be here” [27]. This strong assertion by the speaker expressed that they feel that they owe their life to the facilitation company and that that made their medical trip a successful one. Monetary references varied between videos, depending on whether the testimonial focused on relative cost savings. For example, one video advertised that the patient “saved 7,000 euro, a 50% lower cost compared to the Netherlands” [28].

A second category that emerged included characteristics associated with the service provided, including the quality and availability of the surgeon or other health care professionals who provided the procedure, the quality and friendliness of the support staff, reference to the quality and expediency of care, and any reference to the general cleanliness and/or services provided by the clinic/hospital. The quality of the surgeon was referenced in 17 of the videos analyzed with a total frequency of 42 while the quality of the support staff was discussed in 14 of the videos viewed with a frequency of 22. Reference to the quality and expediency of care was also made in 14 videos, but had significantly more mentions at a total frequency of 34. Finally, references to general cleanliness and the availability of high-tech services at the facilities abroad were made in 16 out of the 20 videos viewed with a total frequency of 32. In most of the videos, these mentions were expressed verbally, but a few of the testimonials took the viewer on a virtual tour of the facilities as a method of portraying the cleanliness and availability of high-tech services. Speaker statements varied greatly between videos with regard to what aspects from this category the speaker chose to focus on. For example, commentary on the medical staff and accommodations included statements such as “everyone was concerned with making my stay in Guatemala a pleasant one” [29]. Another statement focused on the surgeon, proclaiming that the “surgeon spoke very good English, which is extremely important” [30]. A third statement focused on the facilities stating the “clinics are modern…nothing scary” [31].

The final category consisted of referrals for other persons considering engaging in medical tourism. This was evident in 14 of the videos viewed and had a total frequency of 19. The majority of testimonials that contained these referrals made them in a strong and assertive manner. They took the form of encouraging any and all prospective patients to participate in medical tourism and included several variations on the claim that ‘it was the best decision of their life’.

## Discussion

The results of this thematic content analysis have confirmed the findings of the existing medical tourism literature, specifically in the area of medical tourism facilitator webpages and risk communication. A thematic content analysis of Canadian medical tourism facilitator websites conducted by Penney et al. [15] found that risks associated with the advertised procedure were not discussed at all in 47.1% of the websites examined. When risks were addressed in the remaining websites, they were only discussed in a way to showcase how they would not occur if a facilitating agency was used. The majority of the testimonials we examined included no reference to potential risk or harm that may arise from engaging in this practice. If the former patients referenced any fears they had associated with having a medical procedure done in a foreign country, they minimized these fears with mention of the facilitator agency they used and the highly trained medical personnel in the facilities that they accessed that were available to address all of their needs.

### Informed decision-making

The number of testimonials a prospective patient views in support of or against a particular treatment option is an important factor to determining the credibility of the messages being portrayed in patient testimonials [17, 20]. As demonstrated in our findings, the videos supported by medical tourism facilitation companies only portray a positive message of medical tourism as a treatment option. Should those considering medical tourism not be exposed to testimonials of those who did not have a uniformly positive medical tourism experience, prospective patients are more likely to be swayed by the overwhelmingly positive messages portrayed in the testimonials [20]. It is in these facilitation companies’ best interest to display a plethora of positive messages to prospective patients as studies have found that word-of-mouth advertising and personal experiences are some of the major information sources that patients draw from [32]. Companies that choose to employ these advertisement strategies over others also tend to observe “better short- and long-term profits thus, creating sustainable and better growth rates” (pg. 198) [32]. Given that medical tourism facilitators typically are remunerated through referral payments from international clinics rather than directly from clients, they have a financial interest to be able to direct their clients to those clinics with the highest referral payments and for multiple treatments [9]. Viewers of these testimonial videos will typically not be aware of the conflict of interest created by these referral payments, further undermining the capacity of these individuals to make informed decisions about their medical care.

It is problematic for a patient to make a health care decision that is not fully informed. In the patient testimonial videos we examined, content on the benefits of undertaking the given procedure through medical tourism far out-weighed content on the risks of undergoing such procedure, if the risks were discussed at all. Thus, unless patients do additional research beyond the scope of the information provided in the facilitator websites or in the subjective patient testimonial videos, prospective patients may make health care decisions without truly weighing the risks of medical tourism against its benefits [15]. Even if these videos are only one among many information sources used by individuals considering medical tourism, the strong bias presented in these videos, lack of information about the risks of medical tourism, and outsized influence of testimonials on medical decision-making will likely influence behaviour in a way that is contrary to the goal of informed decision-making. Given these implications, patient testimonials can be viewed as morally problematic because testimonials are relied on heavily in decision-making and studies have demonstrated that individuals place much more weight on narrative first-hand accounts of information than other forms of information [17].

### Policy responses

Given the lack of regulation of the medical tourism industry, it is challenging for policy makers to address public health and ethical concerns associated with the existence of medical tourism facilitation webpages and testimonial videos. Information on these webpages and in these videos tends to be incomplete or misleading for medical tourists seeking to gain information before engaging in the practice [33]. One route to addressing this issue would be to enact greater regulation over the medical tourism industry, and especially the facilitation companies. Such a response would be extremely challenging to implement, however, as these medial tourism facilitation companies are based in many different countries [34]. Tighter legislation over the messaging of facilitation companies in one country raises the prospect of these companies simply moving to alternative legal jurisdictions with looser regulations and continuing to advertise their services globally online. Unless an international governing body is established to regulate the practices of medical tourism facilitation companies and what information they are allowed to distribute, effectively regulating the industry is unlikely.

Given that medical tourism facilities are a prominent source of information for persons considering engaging in medical tourism [8], it is essential for governments, professional medical groups, and patient advocates to take steps to combat the existing, industry-focused bias of online information by providing new online sources of information and patient testimonials that present a more objective picture of the risks and ethical issues presented by medical tourism. Some examples of these general guidelines do exist now, including from the US Centers for Disease Control [35] and the Canadian government [36]. These guidelines should be expanded and promoted in order to increase the prevalence and visibility. More engaging methods are needed to inform individuals considering medical tourism about the risks associated with this practice as well [37]. As the outsized role of patient testimonials on medical decision-making cannot be easily changed, the use of testimonials can be co-opted by groups seeking to inform those considering engaging in medical tourism of this practice’s associated risks.

### Limitations

While individuals considering whether to engage in medical tourism have been found to access online resources including facilitator websites [7, 11, 12], less is known about what role patient testimonial videos play in their decision-making and how often these videos are viewed by potential medical tourists. Therefore, while we can say with confidence that these testimonials do play a role in the decision-making of some medical tourists and therefore reinforce a bias toward underrepresenting the risks and ethical issues associated with medical tourism, we cannot say how great this role is. For this reason, more study is needed as to the decision-making process of medical tourists, including the role of patient testimonials.

## Conclusion

This paper has established the themes present in patient testimonial videos supported by medical tourism facilitation webpages through a thematic content analysis of twenty testimonial videos on YouTube. Three main categories of themes were prevalent in the majority of the videos analyzed. These categories included: 1) factors associated with the facilitator, 2) the services provided by the facilitator, and 3) whether the former patient would recommend medical tourism as a viable option to future medical tourists. These videos create serious ethical concerns including the potential to undermine fully informed decision-making by potential medical tourists due to missing or misleading information with regard to the risks entailed by medical tourism. In response, non-industry groups should act to increase the presence and visibility of online sources of information presenting the risks and ethical issues associated with medical tourism, including the use of patient testimonial videos that offer a more realistic portrayal of the experience of accessing medical treatment abroad. Future research should examine the degree to which potential medical tourists access testimonial videos and the degree to which these videos influence their decision-making.

### Endnote

^a^There were three additional subcategories associated with theme four that were identified as 4a, 4b and 4c. This was done because some testimonials explicitly referenced the facilitator company by name, while others a) expressed gratitude, b) referred to the help they received from a facilitator representative/guide and c) that the company took care of all care and travel arrangements.
